# Supplementary material for: Coordinate β-adrenergic inhibition of mitochondrial activity and angiogenesis arrest tumor growth
Source: Nat Commun. 2020 Jul 17;11:3606. doi: 10.1038/s41467-020-17384-1 (PMC7368041; doi:10.1038/s41467-020-17384-1)
Supplement: Supplementary file 1 — Supplementary Information [file 41467_2020_17384_MOESM1_ESM.pdf]

Coordinate β1-adrenergic inhibition of mitochondrial activity and angiogenesis arrest tumor growth

Nuevo-Tapioles et al.

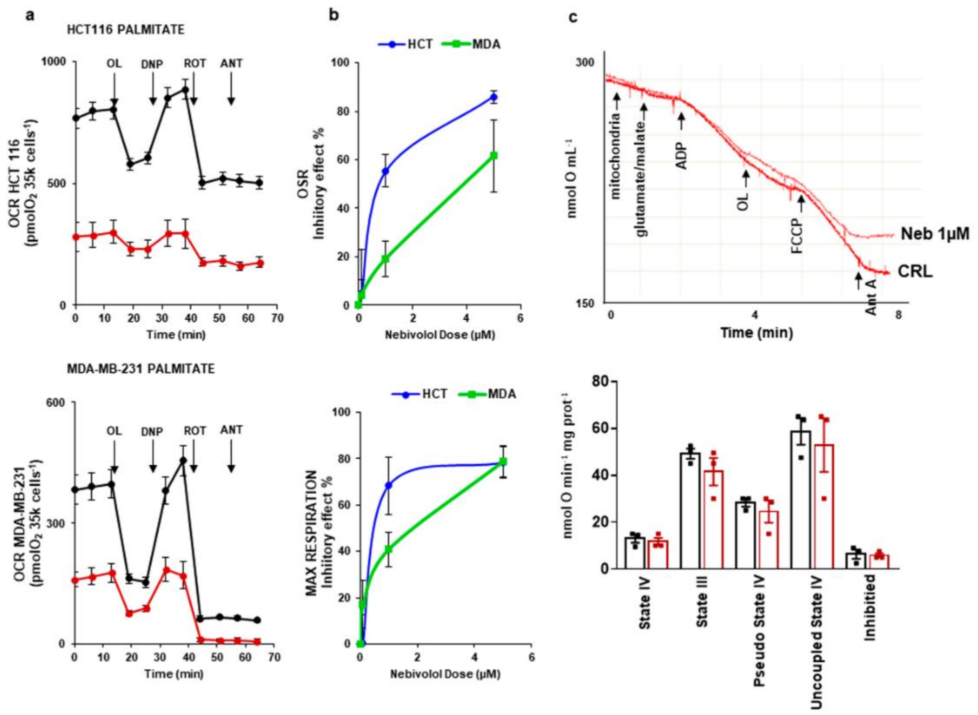

Supplementary Figure 1. Nebivolol inhibits mitochondrial respiration in cancer cells. Related to Figure 1. a Respiratory profile of HCT116 (upper panel) and MDA-MB-231 (lower panel) cells treated (red trace) or not (black trace) for 3 h with nebivolol (1 µM) using palmitate as respiratory substrate. OCR, oxygen consumption rate; OL, oligomycin; DNP, 2,4-dinitrophenol; ROT, rotenone; ANT, antimycin A. **b** Inhibition of oligomycin sensitive respiration (OSR) (upper panel) and maximal respiration (lower panel) at different doses of nebivolol in three biological replicates of HCT116 (purple trace) and MDA-MB-231 (green trace) cells. **c** Polarographic profiles of isolated liver mitochondria treated with nebivolol (1 µM) (upper trace) or left untreated (lower trace). The effect of 10 mM glutamate/malate, 0.5 mM ADP, 6 µM oligomycin (OL), 1 µM FCCP, and 1 µM antimycin A (Ant A) is shown. Histograms show no differences in mitochondrial respiratory states when compared to CRL by two-sided Student's t test. The results are means of three different samples ± SEM. Source data are provided as a Source Data file.

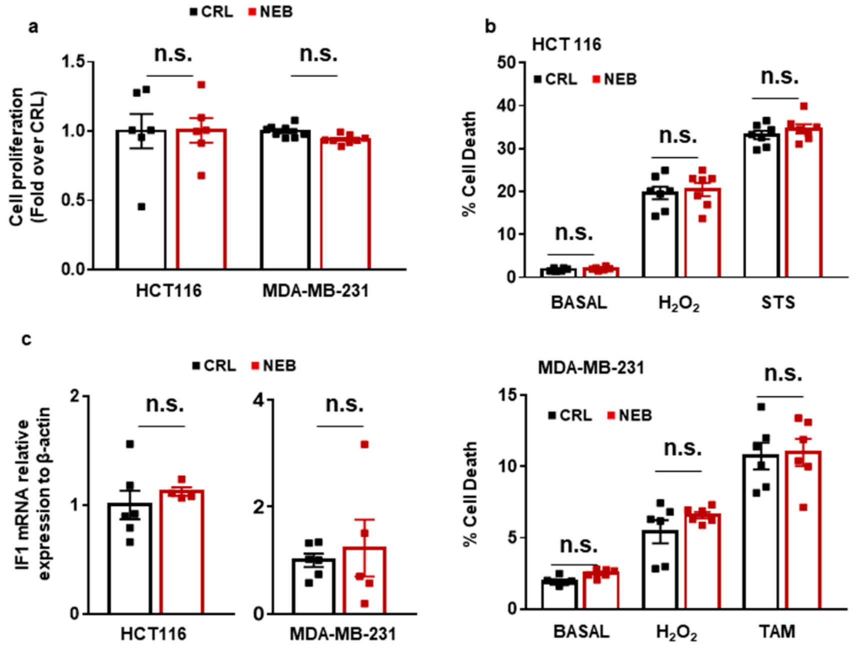

Supplementary Figure 2. Nebivolol does not affect the proliferation and death of cancer cells growing in culture. Related to Figure 2. a-b HCT116 or MDA-MB-231 cells were treated during 48 h with nebivolol (NEB; red bar) or left untreated (CRL; closed bar).

a The histograms show cellular proliferation using the Click-iT EdU Assay Kit. Figure 8a for gating strategy.

b The histograms show cell death responses under basal, 1 µM staurosporine (STS), 120 µM hydrogen peroxide (H_2O_2) or 1 µM tamoxifen (TAM) treatments. See Supplementary Figure 8b for gating strategy.

c Histograms show the quantification by real-time PCR of the mRNA levels encoding IF1 in control (CRL, closed bar) or 3 h nebivolol-treated HCT116 and MDA-MB-231 cells.

Bars indicate the mean ± SEM of three different samples. No differences were found when compared to CRL by two-sided Student's t test. Source data are provided as a Source Data file.

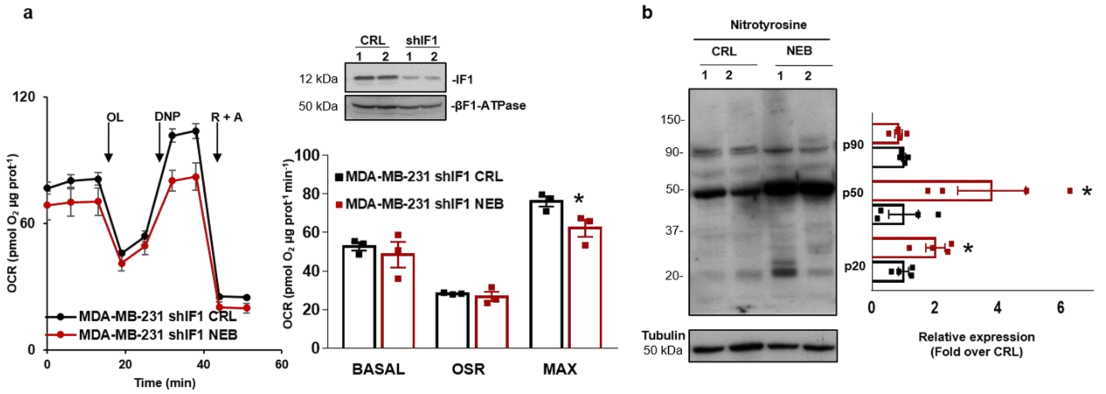

Supplementary Figure 3. Related to Figures 4 and 5

a Respiratory profile and parameters of IF1-silenced MDA-MB-231 cells treated (MDA-MB-231 shIF1 NEB, red trace) or not (MDA-MB-231 shIF1 CRL, black trace) with nebivolol. Basal, oligomycin sensitive respiration (OSR) and maximum (MAX) respiration of three biological replicates are shown in the histograms. OCR, oxygen consumption rate; OL, oligomycin; DNP, 2,4-dinitrophenol; R, rotenone; A, antimycin A. The expression of IF1 and βF1-ATPase subunit of the ATP synthase in a representative western blot of control MDA-MB-231 and IF1 silenced cells is shown in two different samples. *p = 0.05

b Representative western blots of two different samples and quantification of four different samples of nitrotyrosine modified tumor proteins. Tubulin is shown as loading control. *p = 0.05 and 0.02

Bars indicate the mean ± SEM of three (a) or four (b) different samples. *p < 0.05 when compared to CRL by two-sided Student's t test. Source data are provided as a Source Data file.

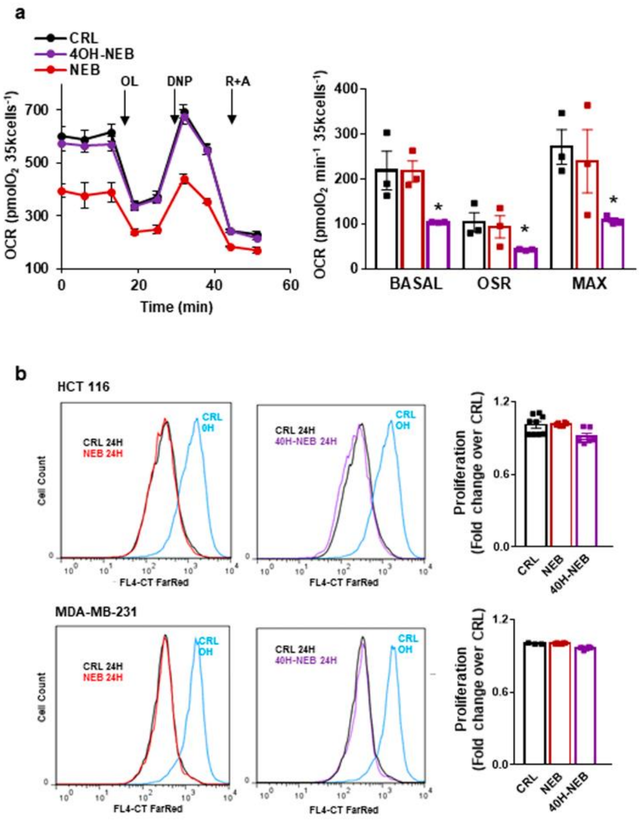

Supplementary Figure 4. 4OH-nebivolol has no effect in respiration and proliferation. Related to Figure 5.

a Respiratory profile and parameters of HCT116 cells treated with nebivolol (red trace), 4OH-nebivolol (purple trace) or left untreated (black trace) for 3 h (1 µM). Basal (*p=0.05), OSR (*p=0.04), oligomycin sensitive respiration; MAX (*p=0.01), maximum respiration; OCR, oxygen consumption rate; OL, oligomycin; DNP, 2,4-dinitrophenol; ROT, rotenone; ANT, antimycin A. **b** The histograms show cellular proliferation in HCT116 (upper panel) and MDA-MB-231 cells (lower panel) treated with nebivolol (red trace), 4OH-nebivolol (purple trace) or left untreated (black trace) after 24 h of CellTrace Red Far incorporation. Blue histogram is shown as time 0 control. See Supplementary Figure 7c for gating strategy.

Bars indicate the mean ± SEM of three different samples. *p < 0.05 when compared to CRL by two-sided Student's t test. Source data are provided as a Source Data file.

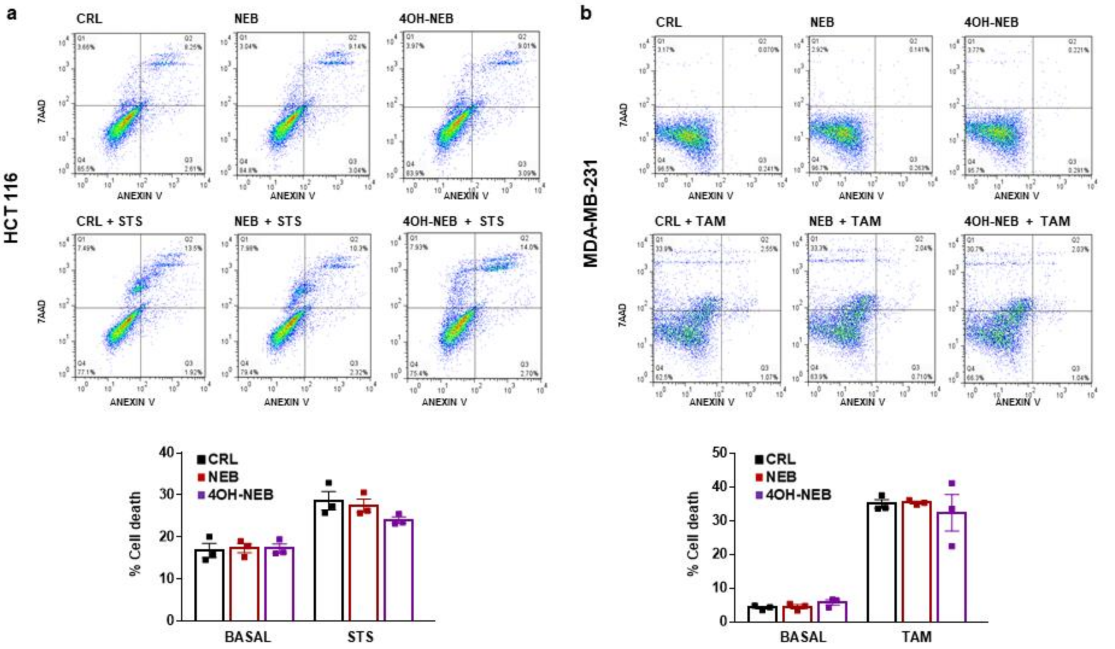

Supplementary Figure 5. 4OH-nebivolol has no effect in cell death. Related to Figure 5.

a-b The plots and histograms show cell death in HCT116 **a** and MDA-MB-231 cells **b** treated with nebivolol (red bar), 4OH-nebivolol (purple bar) or left untreated (closed bar). The histograms show the quantification of basal total cell death and after 24 h treatment with staurosporine (STS) or tamoxifen (TAM).

Bars indicate the mean ± SEM of three different samples. No differences were found when compared to CRL by two-sided Student’s t test. See Supplementary Figure 8b for gating strategy. Source data are provided as a Source Data file.

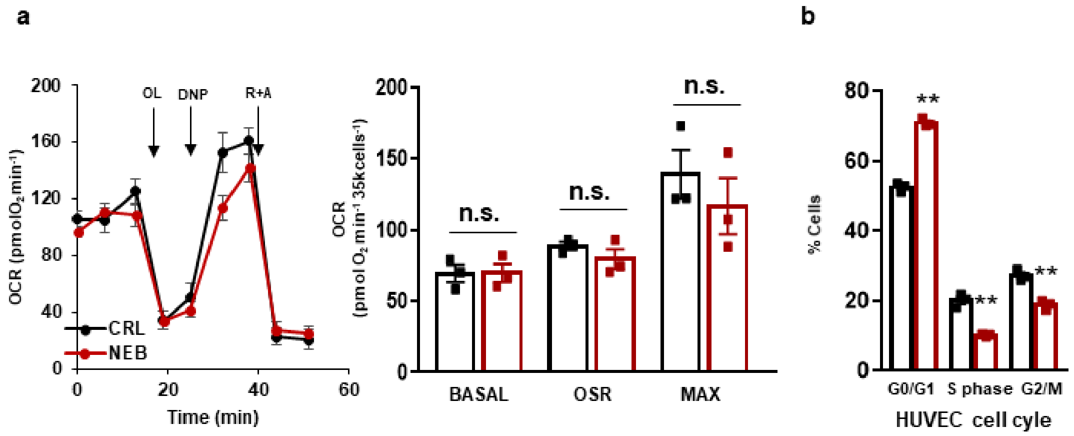

Supplementary Figure 6. Nebivolol arrests endothelial cell cycle in G0/G1 phase. Related to Figure 6.

a Respiratory profile of HUVEC cells treated (NEB, red trace; n=3 different samples) or not (CRL, black trace; n=3 different samples) with nebivolol. Basal, oligomycin-sensitive respiration (OSR) and maximum (MAX) respiration of three biological replicates are shown in the histograms. OCR, oxygen consumption rate; OL, oligomycin; DNP, 2,4-dinitrophenol; R, rotenone; A, antimycin.

b Histograms show the percentage of cells in G0/G1 (**p = 5.2E-05), S (**p = 0.0009) and G2/M (**p = 0.002) phases of the cell cycle in nebivolol-treated (NEB; n=3 different samples) or left untreated (CRL; n=3 different samples) HUVEC cells. See Supplementary Figure 7d for gating strategy. Source data are provided as a Source Data file.

Bars indicate the mean ± SEM of three different samples. **$p < 0.01$ when compared to CRL by two-sided Student's t test. Source data are provided as a Source Data file.

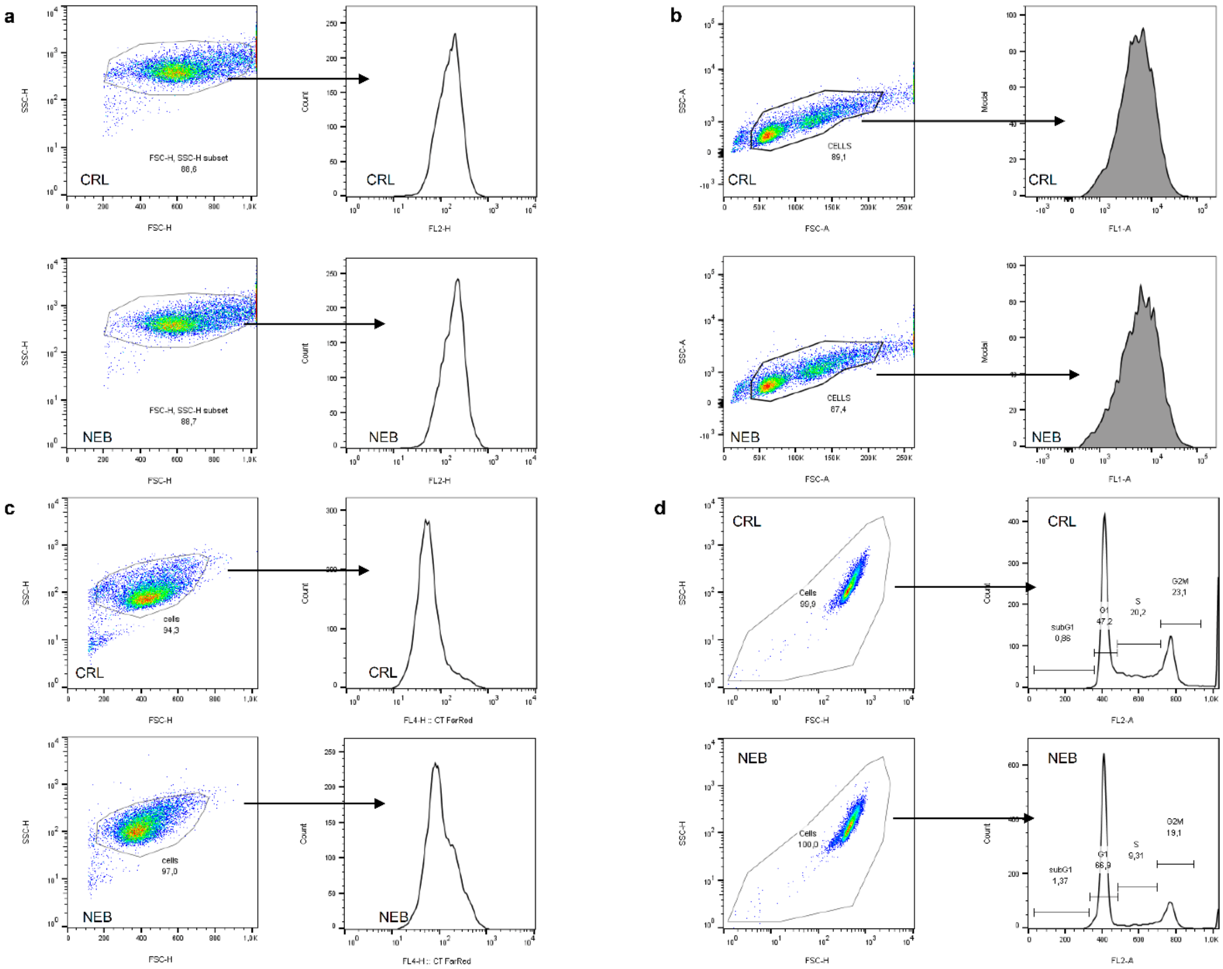

Supplementary Figure 7. Flow cytometry data: Gating strategies.

a Gating strategy to measure membrane potential presented on Fig. 2c. **b** Gating strategy to measure ROS production presented on Fig. 2d. **c** Gating strategy to measure cellular proliferation presented on Fig. 6d and Supplementary Fig. 4b. **d** Gating strategy to measure cell cycle presented on Fig. 6g and Supplementary Fig. 6b.

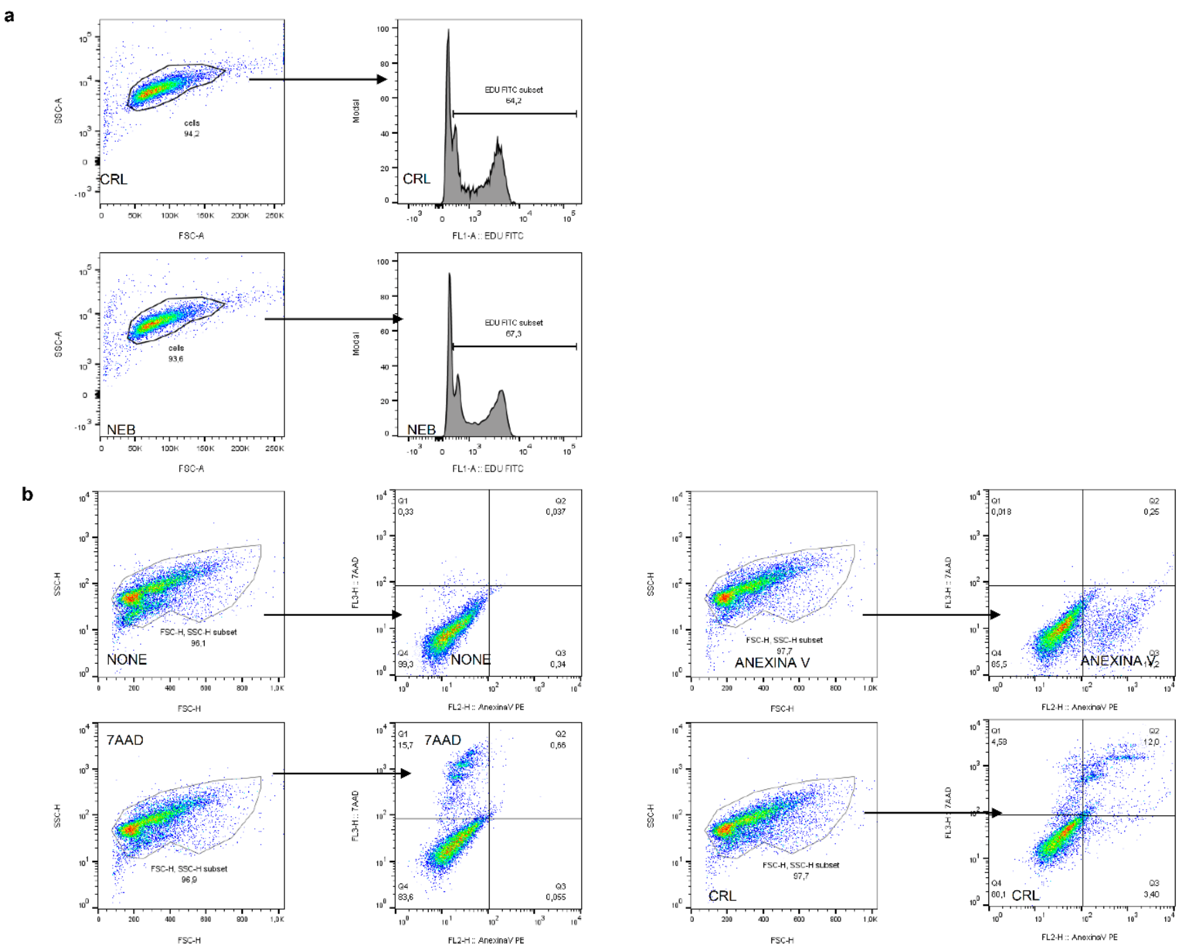

Supplementary Figure 8. Flow cytometry data: Gating strategies.

a Gating strategy to measure cellular proliferation presented on Supplementary Fig. 2a. **b** Gating strategy to measure cell death presented on Supplementary Fig. 2b and Supplementary Fig. 5a,b.
