# Supplementary material for: Coordinate β-adrenergic inhibition of mitochondrial activity and angiogenesis arrest tumor growth
Source: Nat Commun. 2020 Jul 17;11:3606. doi: 10.1038/s41467-020-17384-1 (PMC7368041; doi:10.1038/s41467-020-17384-1)
Supplement: Supplementary file 3 — Reporting Summary [file 41467_2020_17384_MOESM3_ESM.pdf]

nature research

Corresponding author(s): José M. Cuezva

Last updated by author(s): Jun 17, 2020

Reporting Summary

Nature Research wishes to improve the reproducibility of the work that we publish. This form provides structure for consistency and transparency in reporting. For further information on Nature Research policies, see our Editorial Policies and the Editorial Policy Checklist.

Statistics

For all statistical analyses, confirm that the following items are present in the figure legend, table legend, main text, or Methods section.

n/a	Confirmed	
☐	☒	The exact sample size (*n*) for each experimental group/condition, given as a discrete number and unit of measurement
☐	☒	A statement on whether measurements were taken from distinct samples or whether the same sample was measured repeatedly
☐	☒	The statistical test(s) used AND whether they are one- or two-sided<br>*Only common tests should be described solely by name; describe more complex techniques in the Methods section.*
☒	☐	A description of all covariates tested
☒	☐	A description of any assumptions or corrections, such as tests of normality and adjustment for multiple comparisons
☐	☒	A full description of the statistical parameters including central tendency (e.g. means) or other basic estimates (e.g. regression coefficient) AND variation (e.g. standard deviation) or associated estimates of uncertainty (e.g. confidence intervals)
☐	☒	For null hypothesis testing, the test statistic (e.g. *F*, *t*, *r*) with confidence intervals, effect sizes, degrees of freedom and *P* value noted<br>*Give P values as exact values whenever suitable.*
☒	☐	For Bayesian analysis, information on the choice of priors and Markov chain Monte Carlo settings
☒	☐	For hierarchical and complex designs, identification of the appropriate level for tests and full reporting of outcomes
☒	☐	Estimates of effect sizes (e.g. Cohen's *d*, Pearson's *r*), indicating how they were calculated

Our web collection on statistics for biologists contains articles on many of the points above.

Software and code

Policy information about availability of computer code

Data collection	We used Perkin Elmer In vivo imaging (IVIS) system to collect the HCT116-luc images of the tumors; Bio-Rad GS-900 for collecting WB data; BD FACScan to collect flow cytometry data; Nikon A1R + and Zeiss LSM 710 to collect confocal microscopy images
Data analysis	Images analysis were performed using ImageJ software version 1.46. Flow cytometry was analysed using FlowJo software v10.6.2. Perkin Elmer Living Image Software 3.2. was used to analysed IVIS images. All of the statistical analysis were performed by Excel Microsoft 365 and GraphPad Prism 7. Kaplan-Meier curves were analised using IBM SPSS statistics 24.

For manuscripts utilizing custom algorithms or software that are central to the research but not yet described in published literature, software must be made available to editors and reviewers. We strongly encourage code deposition in a community repository (e.g. GitHub). See the Nature Research guidelines for submitting code & software for further information.

Data

Policy information about availability of data

All manuscripts must include a data availability statement. This statement should provide the following information, where applicable:

- Accession codes, unique identifiers, or web links for publicly available datasets
- A list of figures that have associated raw data
- A description of any restrictions on data availability

The mass spectrometry proteomics data have been deposited to the ProteomeXchange Consortium via the PRIDE partner repository with the dataset identifier PXD019750 and 10.6019/PXD019750. The source data underlying Figs 1c-j, 2a-e, 3a, b, 4a-j, 5a-m, 6a-h, 7a-d, 8a-l and Supplementary Figs 1a-c, 2a-c, 3a-b, 4a, b, 5a, b, 6a, b are provided as a Source Data file. All the other data supporting the findings of this study are available within the article and its supplementary information files, and from the corresponding author upon reasonable request. A reporting summary for this article is available as a Supplementary Information file.
Source data are provided with this paper.

Field-specific reporting

Please select the one below that is the best fit for your research. If you are not sure, read the appropriate sections before making your selection.

☒ Life sciences ☐ Behavioural & social sciences ☐ Ecological, evolutionary & environmental sciences

For a reference copy of the document with all sections, see nature.com/documents/nr-reporting-summary-flat.pdf

Life sciences study design

All studies must disclose on these points even when the disclosure is negative.

Sample size	Required sample sizes were determined based on experience from previous experiments performed in our laboratory which reached statistically relevant results as shown in Sánchez-Aragó Carcinogenesis 2010; EMBO Rep. 2013; Formentini et al., EMBO J. 2014; González-Llorente et al., Cancers, 2020; Santacatterina et al., Oncotarget, 2016; García-Bermúdez et al., Cell Reports, 2015.
Data exclusions	No data were excluded
Replication	All of the experiments were repeated for at least 3 times. All the attempts were successful.
Randomization	In the xenograft models, when HCT116 tumors reached ~ 100 mm3 of volume, animals were randomly allocated into different groups. In the ortothopic model, after obtaning the same bioluminescence detection of a stable signal, mice were randomly allocated into different groups. Randomization was not relevant for other experiments, as they were performed in cell lines.
Blinding	Investigators were blinded during in vivo IVIS adquisition and analysis and tumor samples immunofluorescence analysis. Investigators were not blinded during in vitro treatment, data collection or analysis because the sample names contained treatment information to allow the identification.

Reporting for specific materials, systems and methods

We require information from authors about some types of materials, experimental systems and methods used in many studies. Here, indicate whether each material, system or method listed is relevant to your study. If you are not sure if a list item applies to your research, read the appropriate section before selecting a response.

Materials & experimental systems

n/a	Involved in the study	
☐	☒	Antibodies
☐	☒	Eukaryotic cell lines
☒	☐	Palaeontology and archaeology
☐	☒	Animals and other organisms
☒	☐	Human research participants
☒	☐	Clinical data
☒	☐	Dual use research of concern

Methods

n/a	Involved in the study	
☒	☐	ChIP-seq
☐	☒	Flow cytometry
☒	☐	MRI-based neuroimaging

Antibodies

Antibodies used	The primary antibodies used were anti-human IF1 (clone 14/2 generated and provided by Cuezva's lab, 1:100), anti-β-F1-ATPase (clone 11/21-7A8 generated and provided by Cuezva's lab, 1:1,000), anti-GAPDH (1:20000, Abcam, ab8245), anti-NDUFA9 (1:1000, Abcam, ab14713), anti-NDUFS3 (1:1000, Abcam, ab14711) anti-SDH-B (1:500, Invitrogen,21A11AE7), anti-Core 2 (1:500, Abcam, ab14745), anti-COX4 (1:1000, Abcam, ab14744), anti-Hsp60 (Stressgene, 1:2000, ADI-SPA-807-E), anti-phosphoserine (1:100, Sigma, P5747), anti-nitrotyrosine (1:1000, Abcam, ab61392), anti-glutathione reductase (1:1000, Santa Cruz Biotechnology, sc-133245), anti-α-tubulin (1:1000, Sigma, T5168), anti-catalase (1:20000, Sigma, C0979), anti-SOD2 (1:1000, Abcam, sc-137254) anti-PRx6 (1:1000, Abcam, ab59543), anti-PRx3 (1:1000, Invitrogen, ab222807), anti-G6PDH (1:1000, Cell signaling, #8866), anti-VDAC (1:500, Abcam, ab15895). anti-ADRB1 (1:500, Santa Cruz Biotecnologies, sc-567), anti-VEGF (1:1000, Abcam, ab46154), anti-VEGFR2 (1:1000, Cell signaling, #2479), anti-phosphoERK (1:500, Cell Signaling, #9101) and anti-ERK (1:500, Santa Cruz Biotechnologies, sc-514302). Secondary antibodies used: Peroxidase-conjugated anti-mouse (1:3000, Promega, W4021) or anti-rabbit (1:3000, Promega, W4011) IgGs. For angiogenesis determination: fluorescein labeled GSL I-B4 (5 μg/ml, Vector laboratories, FL-1201), anti-CD31 (1:200, BD Pharmingen, 553370), anti-Actin, α-Smooth Muscle - Cy3 (1:100, Sigma, C6198) and anti-Laminin (1:25, Sigma, L9393) anti-activated caspase-3 (1:200, Cell signaling, #9661) to assess cell-death and anti-Ki67 (1:250, Thermo Fisher Scientific, RM-9106)
Validation	Anti-human IF1 (clone 14/2, 1:100)15, anti-β-F1-ATPase (clone 11/21-7A8, 1:1,000) were produced in the laboratory and validated in Sanchez-Cenizo , J Biol Chem 285, 25308-25313 (2010) and Acebo P, Transl Oncol 2, 138-145 (2009), respectively. The other antibodies are commercially available and validated in the literature as cited on the manifacturer's websites, as well as by

the datasheet they provide:
anti-GAPDH: https://www.abcam.com/gapdh-antibody-6c5-loading-control-ab8245.html
anti-NDUFA9: https://www.abcam.com/ndufa9-antibody-20c11b11b11-ab14713.html
anti-NDUFS3: https://www.abcam.com/ndufs3-antibody-17d95-ab14711.html
anti-SDH-B: https://www.thermofisher.com/antibody/product/SDHB-Antibody-clone-21A11AE7-Monoclonal/459230
anti-Core 2: https://www.abcam.com/uqcrc2-antibody-13g12af12bb11-ab14745.html
anti-COX4: https://www.abcam.com/cox-iv-antibody-20e8c12-ab14744.html
anti-Hsp60: https://www.enzolifesciences.com/ADI-SPA-807/hsp60-monoclonal-antibody-lk-2/
anti-phosphoserine: https://www.sigmaaldrich.com/catalog/product/sigma/p5747?lang=es&region=ES
anti-nitrotyrosine: https://www.abcam.com/3-nitrotyrosine-antibody-39b6-ab61392.html
anti-glutathione reductase: https://www.scbt.com/es/p/glutathione-reductase-antibody-c-10
anti-α-tubulin: https://www.sigmaaldrich.com/catalog/product/sigma/t5168?lang=es&region=ES
anti-catalase: https://www.sigmaaldrich.com/catalog/product/sigma/c0979?lang=es&region=ES
anti-SOD2: https://www.scbt.com/es/p/sod-2-antibody-e-10
anti-PRx6: https://www.abcam.com/peroxiredoxin-6-antibody-ab59543.html
anti-PRx3: https://www.abcam.com/peroxiredoxin-3prdx3-antibody-ab222807.html
anti-G6PDH: https://www.cellsignal.com/products/primary-antibodies/glucose-6-phosphate-dehydrogenase-antibody/8866
anti-VDAC: https://www.abcam.com/vdac1-porin-antibody-mitochondrial-loading-control-ab15895.html
anti-VEGF: https://www.abcam.com/vegfa-antibody-ab46154.html
anti-VEGFR2: https://www.cellsignal.com/products/primary-antibodies/vegf-receptor-2-55b11-rabbit-mab/2479
anti-phosphoERK: https://www.cellsignal.com/products/primary-antibodies/phospho-p44-42-mapk-erk1-2-thr202-tyr204-antibody/9101
Anti-Mouse IgG Antibody, Peroxidase Conjugated, H+L: https://www.promega.es/products/protein-detection/primary-and-secondary-antibodies/anti_mouse-igg-h-and-l-hrp-conjugate/?catNum=W4021
Anti-Rabbit IgG Antibody, Peroxidase Conjugated, H+L: https://www.promega.es/products/protein-detection/primary-and-secondary-antibodies/anti-rabbit-igg-h-and-l-hrp-conjugate/?catNum=W4011
anti-ERK: https://www.scbt.com/es/p/erk-1-2-antibody-c-9
fluorescein labeled GSL I-B4: https://vectorlabs.com/fluorescein-labeled-gsl-i-isolectin-b4.html
anti-CD31: https://www.bdbiosciences.com/eu/applications/research/stem-cell-research/cancer-research/mouse/purified-rat-anti-mouse-cd31-mec-133/p/553370
anti-Actin, α-Smooth Muscle - Cy3: https://www.sigmaaldrich.com/catalog/product/sigma/c6198?lang=es&region=ES
anti-Laminin (1:25, Sigma): https://www.sigmaaldrich.com/catalog/product/sigma/l9393?lang=es&region=ES
anti-activated caspase-3: https://www.cellsignal.com/products/primary-antibodies/cleaved-caspase-3-asp175-antibody/9661
anti-Ki67: https://www.thermofisher.com/order/catalog/product/RM-9106-R7#/RM-9106-R7

Eukaryotic cell lines

Policy information about cell lines

Cell line source(s)	HCT116-luc were obtained from Caliper Life Sciences, Inc. (Hopkinton, MA, USA). HUVEC cells were kindly provided by Dr. Jaime Millán (CBMSO, Madrid) and obtained from umbilical cords of normal deliveries (after approval by the ethics committee of the Hospital "Ruber Internacional"). HCT116, MDA-MB-231, SH-SY5Y, A549, C2C12, OVCAR8, NRK and Hs 578T cell lines were obtained from ATCC (Manassas, VA, USA). HCT116-shIF1 and MDA-MB-231-shIF1 cells were developed in our laboratory from the original ATCC HCT116 and MDA-MB-231.
Authentication	Human cell lines were authenticated by STR-(microsatellite) genotyping by the Genomic Unit at Instituto de Investigaciones Biomédicas (CSIC-UAM).<br>Animal cell lines were not authenticated.
Mycoplasma contamination	All cell lines were tested as negative for mycoplasma contamination.
Commonly misidentified lines<br>(See ICLAC register)	There are no misidentified cell lines in this study.

Animals and other organisms

Policy information about studies involving animals; ARRIVE guidelines recommended for reporting animal research

Laboratory animals	We have used 6 weeks, male (HCT116 experiments) and female (MDA-MB-231 experiments) Athymic Nude Mice. A 12-hour light/12-hour dark cycle. Temperatures of 18-23°C with 40-60% humidity.
Wild animals	There are no wild animals used in this study.
Field-collected samples	There are no field-collected samples used in this study.
Ethics oversight	The Ethics Committee of Animal Experimentation (CSIC-UAM, CM PROEX 023/14) and the Institutional Review Board of UAM (CEI 75-1365) approved the project.

Note that full information on the approval of the study protocol must also be provided in the manuscript.

Flow Cytometry

Plots

Confirm that:

☒ The axis labels state the marker and fluorochrome used (e.g. CD4-FITC).

☒ The axis scales are clearly visible. Include numbers along axes only for bottom left plot of group (a 'group' is an analysis of identical markers).

☒ All plots are contour plots with outliers or pseudocolor plots.

☒ A numerical value for number of cells or percentage (with statistics) is provided.

Methodology

Sample preparation	For the determination of the mitochondrial membrane potential, 300,000 cells were incubated with 0.5 µM TMRM+ in 300 µl of FACS (1mM EDTA, 2%FSB in PBS) and processed for flow cytometry to determine the mitochondrial membrane potential with 1 µM nebivolol, 5 µM FCCP, 6 µM oligomycin or left untreated.<br>Cellular proliferation was determined by the incorporation of 5-ethynyl-20deoxy-uridine (EdU) into cellular DNA using the Click-iT EdU Flow Cytometry Assay Kit (Thermo Fisher Scientific) and using CellTraceTM Far Red (Thermo Fisher Scientific). Cells were incubate with the dye for 20 minutes at 37°C and wash twice with culture medium containing at least 1% protein before analysis.<br>For cell cycle analysis, cells were trypsinized, centrifuged at 1,000 × g for 5 min, collected and washed with ice-cold PBS. Cellular pellets were resuspended and fixed with cold 70% ethanol overnight. After another wash with PBS, the cell pellets were resuspended in 1 ml of staining solution containing propidium iodide (PI, 50 µg/ml). Finally, the cells were incubated at 37°C for 30 min in the dark before analysis.<br>For cell death assays, cells were seeded and treated with 1 µM staurosporine (STS), 120 µM hydrogen peroxide (H2O2) or 1 µM tamoxifen as indicated. Cell death was determined by flow cytometry after staining with annexin V and 7-AAD (Annexin V Apoptosis Detection Kit I, BD PharmingenTM).<br>The intracellular production of hydrogen peroxide was monitored by flow cytometry using 10µM 6-carboxy-2',7'-dichlorodihydrofluorescein diacetate (DCFH2-DA) (Thermo Fisher Scientific). Cells were removed and pre-warmed in HBSS buffer containing the dye for 30 min. Cells were washed twice before analysis.
Instrument	The instrument used for data collection was a FACSCalibur (BD)
Software	Software Data collection was performed with the Cell Quest-PRO software and data analysis was done using FlowJo v10 cytometry Software
Cell population abundance	In all cases 10,000 events were recorded to measure mitochondrial membrane potential, cellular proliferation, cell cycle, ROS production and cell death.
Gating strategy	Debris were removed from the main cell population using the FSC/SSC gating. The positive population for each sample was defined on the basis of the signal of unstained cells.<br>In case of double-staining, gating parameters were set using different samples including untreated and 4OH-NEB-treated cells, and cells incubated only with Annexin V or 7AAD.

☒ Tick this box to confirm that a figure exemplifying the gating strategy is provided in the Supplementary Information.
